# Awake chronic mouse model of targeted pial vessel occlusion via photothrombosis

**DOI:** 10.1117/1.NPh.7.1.015005

**Published:** 2020-01-30

**Authors:** Smrithi Sunil, Sefik Evren Erdener, Blaire S. Lee, Dmitry Postnov, Jianbo Tang, Sreekanth Kura, Xiaojun Cheng, Ichun Anderson Chen, David A. Boas, Kıvılcım Kılıç

**Affiliations:** a Boston University, Neurophotonics Center, Department of Biomedical Engineering, Boston, Massachusetts, United States; b Hacettepe University, Institute of Neurological Sciences and Psychiatry, Ankara, Turkey; c Copenhagen University, Department of Biomedical Sciences, Copenhagen, Denmark

**Keywords:** stroke, photothrombosis, imaging, awake, chronic

## Abstract

Animal models of stroke are used extensively to study the mechanisms involved in the acute and chronic phases of recovery following stroke. A translatable animal model that closely mimics the mechanisms of a human stroke is essential in understanding recovery processes as well as developing therapies that improve functional outcomes. We describe a photothrombosis stroke model that is capable of targeting a single distal pial branch of the middle cerebral artery with minimal damage to the surrounding parenchyma in awake head-fixed mice. Mice are implanted with chronic cranial windows above one hemisphere of the brain that allow optical access to study recovery mechanisms for over a month following occlusion. Additionally, we study the effect of laser spot size used for occlusion and demonstrate that a spot size with small axial and lateral resolution has the advantage of minimizing unwanted photodamage while still monitoring macroscopic changes to cerebral blood flow during photothrombosis. We show that temporally guiding illumination using real-time feedback of blood flow dynamics also minimized unwanted photodamage to the vascular network. Finally, through quantifiable behavior deficits and chronic imaging we show that this model can be used to study recovery mechanisms or the effects of therapeutics longitudinally.

## 1 Introduction

Stroke is the leading cause of long-term disability and the second leading cause of death worldwide.^1^ Strokes are either ischemic, which account for over 85% of all strokes and occur due to the interruption of blood flow caused by thrombosis, or hemorrhagic, which occur due to bleeding.^2^^,^^3^ During ischemic stroke, a reduction or complete loss in blood supply causes a starved oxygen environment and leads to neuronal damage within minutes and ultimately to sensorimotor and cognitive impairment.^2^^,^^3^

Animal models provide a great tool to study the structural and functional consequences of ischemic stroke as well as to develop therapeutic methods to promote recovery after stroke.^4^^–^^6^ In order to maximize the translation of preclinical research to the clinical setting, it is essential that animal models of stroke mimic the biology of human stroke.^5^^,^^7^^–^^9^ The most commonly used animal model of ischemic stroke is occlusion of the middle cerebral artery (MCA), which is usually induced by direct mechanical blocking of blood flow via a suture inserted through the carotid artery.^10^^,^^11^ One of the disadvantages of this model is the use of anesthetics in order to perform the occlusion as anesthetics are potential confounding factors that alter neuronal function and cerebral blood flow (CBF).^12^^–^^14^ Another drawback of this technique is the difficulty in monitoring changes to blood flow during occlusion since the animal is on its back during the procedure.^11^ Although a recent study has shown the ability to perform MCA occlusion in a semiawake setting, the animal preparation is acute and does not allow longitudinal monitoring of stroke progression.^15^ Other currently used models of ischemic stroke are through ferric chloride–induced vascular thrombosis,^16^ endothelin-1–induced vasoconstriction,^17^ or thrombin injection.^18^ However, these models also suffer from the drawbacks of using anesthetics, being acute preparations, and requiring invasive surgery without recovery to induce stroke.

Another common model of ischemic stroke is photothrombosis, which induces ischemic stroke by photoactivation of a photosensitive dye.^19^ Illumination of the injected dye with a specific wavelength of light triggers a clotting cascade and leads to thrombosis. An advantage of this method is that it can be performed in an awake animal, eliminating anesthetics as a confounding factor.^20^ Additionally, simultaneous imaging of CBF changes or other physiological parameters such as tissue oxygen can be performed during photothrombosis, which can further our understanding of the mechanisms involved during stroke.^21^ Photothrombosis also offers the benefit of targeting a specific region by selectively illuminating a region of interest, such as the forelimb region or the barrel cortex in rodents, to study the functional consequences of stroke on specific behaviors over weeks and even months.^22^^–^^24^ However, a disadvantage of illuminating a functionally distinct cortical area is the nonphysiological nature of photocoagulating an area of the brain.^25^ This technique of direct cortical illumination leads to widespread thrombosis of all vasculature within the illumination field, including arteries, arterioles, venules, and capillaries. In a natural situation, only the artery supplying the field is occluded. To maximize the relevance of animal models to human stroke, it is crucial to target individual vessels and minimize unwanted photoactivation-induced damage to the tissue surrounding the target vessel. Previous studies, albeit under anesthesia, have shown the ability to occlude surface arterioles or individual penetrating arterioles using photothrombosis by controlling the parameters of laser illumination in order to minimize photodamage to adjacent and deeper vessels.^26^^–^^28^

Although photothrombosis has been performed in awake animals^29^ and has also been used to target individual vessels,^26^ these methods have not been previously combined. Here we combine the benefits of formerly used techniques to create a chronically prepared mouse photothrombosis stroke model that induces targeted ischemic stroke in awake mice while simultaneously monitoring macroscopic changes to CBF. We show the benefits of targeted illumination through Monte Carlo simulations as well as the benefits of guiding illumination using real-time feedback of CBF through *in vivo* measurements. As shown through laser speckle contrast imaging (LSCI) and behavioral evaluation, this method leads to the formation of a reliable stroke with a functional deficit, which can be monitored chronically to study recovery mechanisms following stroke or to test the effects of potential therapeutics on the recovery process.

## 2 Methods

### 2.1 Instrumentation

**Fig. 1:**
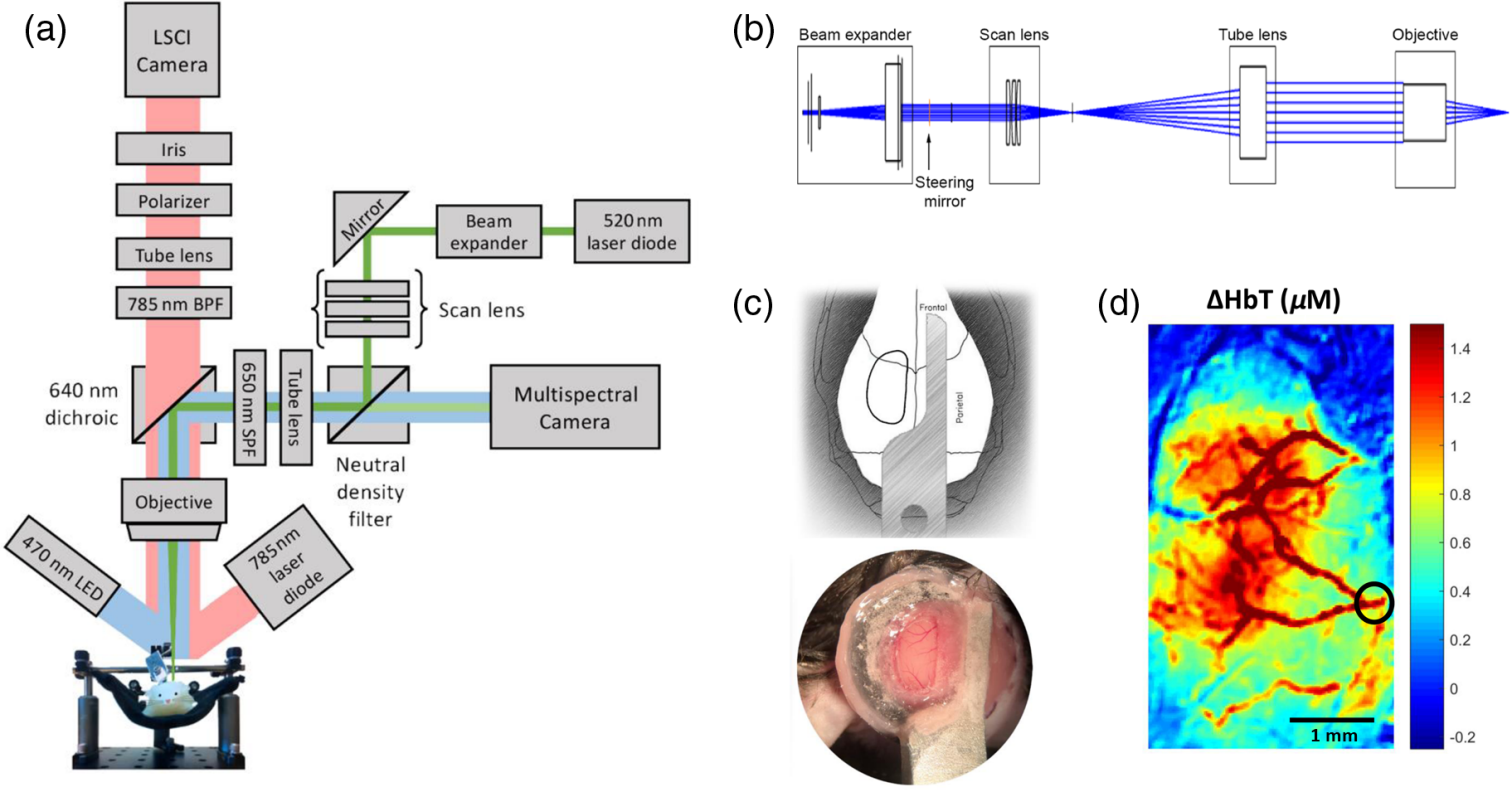
(a) Schematic of imaging setup with combined LSCI, intrinsic signal imaging, and photothrombosis. A parts list for this setup is included in Table S2 in the Supplementary Material. (b) Zemax model of photothrombosis via epi-illumination. (c) (top) Illustration of cranial window and head-bar placement; (bottom) representative image of cranial window and head-bar immediately after surgery. (d) Intrinsic optical signal imaging of change in total hemoglobin concentration during air puff stimulation of the contralateral forelimb. Black circle indicates the vessel targeted for photothrombotic occlusion.

A schematic of the imaging system used for photothrombotic stroke is shown in Fig. 1(a). To induce an occlusion in a distal pial branch of the MCA and simultaneously monitor changes to CBF, the photothrombosis setup was coupled together with a laser speckle imaging system. The photothrombotic arm of the setup consists of a 520-nm laser diode (L520P50, 50 mW, Thorlabs) to photoactivate Rose Bengal via epi-illumination. A scan lens, which consists of three plano-convex lenses and tube lens combination, as shown in Fig. 1(b), was used to fill the back pupil of a 2× objective in order to obtain a focal spot with a lateral resolution of 6  μm in diameter and an axial point spread function of 104  μm. A neutral density filter along the imaging path allowed visualization of the illumination spot through a multispectral camera by avoiding oversaturation of the camera (Hamamatsu ORCA-Flash4.0 V3). By illuminating the cranial window with a blue light emitting diode (LED), the surface vasculature can also be visualized through the multispectral camera, thus making it possible to image the vasculature while performing photothrombosis. A 650-nm short-pass filter was placed along the path to prevent bleed through from the 785-nm laser diode used for laser speckle imaging. Simultaneous LSCI was performed by illuminating the cranial window using a VHG stabilized 785-nm laser diode^30^ (LP785-SAV50, Thorlabs) at a power density of $∼10\;{\mathrm{mW}/\mathrm{cm}}^{2}$ to provide an optimal contrast range and signal-to-noise ratio.^31^ The back scattered light was imaged onto a CMOS camera (Basler acA2040-90  μm NIR, 2048×2048  pixels, 5.5×5.5  μm pixel size) with a 2× magnification for a field of view of 5.6  mm×5.6  mm. A 640-nm dichroic was used to split the visible and near-infra-red illumination. A polarizer and iris in the imaging path were used to reduce specular reflection and optimize for the speckle size, respectively. Images were acquired at 40 frames per second with a 5-ms exposure. Real-time spatial laser speckle contrast was calculated from the raw images captured using software from the Functional Optical Imaging Laboratory at the University of Texas at Austin.^32^

### 2.2 Surgical Procedure

All animal procedures were approved by the Boston University Institutional Animal Care and Use Committee and were conducted following the Guide for the Care and Use of Laboratory Animals. All mice used in this study were adult WT C57Bl6 at ∼12 weeks old during the time of surgery. There were two groups of three mice each to validate our optimized and nonoptimized photothrombotic stroke protocols, three mice were used to assess behavioral changes after stroke, and an additional three mice were used for histological analysis.

Mice were anesthetized using isoflurane (3% induction, 1% to 1.5% maintenance, in 1  L/min oxygen). Dexamethasone was administered 4 h prior to the start of the procedure to minimize tissue swelling during surgery. Body temperature was maintained at 37°C and mice were monitored using respiratory rate and toe pinch throughout the procedure. A craniotomy was performed over one hemisphere of the brain and the skull was removed leaving the dura intact. The surface was covered with a half-skull-shaped curved glass^33^ (modified from Crystal Skull, LabMaker, Germany) and sealed with dental acrylic. An aluminum bar was attached to the intact skull on the other hemisphere for head fixing the mice during photothrombosis and imaging. Animals were given buprenorphine and a prophylactic dose of cefazolin to reduce pain and risk of infection following surgery. The mice were allowed to recover from surgery for at least 10 days before head fixation. Mice were trained to become accustomed to head fixation for up to 1 h for ∼10  days before performing photothrombosis. All experiments are done in awake head-fixed mice.^34^^–^^36^ A cranial window and head-bar illustration and preparation is shown in Fig. 1(c).

### 2.3 Intrinsic Optical Signal Imaging

In order to determine the target vessel for photothrombotic occlusion, we performed intrinsic optical signal imaging during air puff sensory stimulation of the contralateral forelimb. Awake head-fixed mice were placed under the imaging setup shown in Fig. 1(a). Three LEDs with center wavelengths at 470, 530, and 625 nm (MXL3-C1, Thorlabs, X is the center wavelength) were used to sequentially illuminate the cranial window at 30 Hz (10 Hz per wavelength). Images were collected by the sCMOS camera (Hamamatsu ORCA-Flash4.0 V3). For sensory stimulation, each trial consisted of a 5-s train of air puffs at 3 Hz delivered to the forelimb contralateral to the hemisphere implanted with the cranial window. We obtained 20 trials where each trial was obtained in a block-design fashion and consisted of 5 s of baseline, followed by 5 s of stimulation, followed by 20 s of recovery. A custom MATLAB code was used to synchronize and trigger the sequential LEDs, camera acquisition, and air puff stimulation.

We computed changes in oxy-hemoglobin (HbO) and deoxy-hemoglobin (HbR) from the acquired images using the modified Beer–Lambert relationship. Briefly, from the sequence of images acquired, we computed the change in optical density (OD) at each wavelength for each pixel as

$$\Delta \mathrm{OD}(\lambda ,t)=-\ln[\frac{I(\lambda ,t)}{I_{0}(\lambda )}],$$

where I(λ,t) is the reflected light intensity at wavelength λ and time t, and $I_{0}(\lambda )$ is the baseline intensity (first 5 s of every trial). Using the modified Beer–Lambert law:

$$I(\lambda ,t)=I_{0}(\lambda )\exp[-\Delta \mu_{a}(\lambda ,t)L(\lambda )],$$

where $\Delta \mu_{a}(\lambda ,t)$ is the change in optical absorption coefficient and L(λ) is the wavelength-dependent mean free path length in the tissue as estimated by Kohl et al.^37^ through Monte Carlo simulations of light propagation in tissue, we estimated $\Delta \mu_{a}(\lambda ,t)$ at each wavelength for each pixel. Using the estimated changes in absorption coefficients, we calculated changes in HbO and HbR for each pixel at each time using a set of three equations and the molar extinction coefficients of HbO and HbR:

$$\Delta \mu_{a}(\lambda ,t)=\epsilon_{\mathrm{HbO}}(\lambda )\Delta \mathrm{HbO}(t)+\epsilon_{\mathrm{HbR}}(\lambda )\Delta \mathrm{HbR}(t).$$

Total hemoglobin HbT was calculated as a sum of HbO and HbR. HbT for 20 trials was averaged and the spatial map for change in HbT was obtained for the 5-s stimulus period. This spatial map was used to determine the branch of the MCA that supplied the forelimb region, which was the target branch for photothrombotic occlusion. Figure 1(d) shows a representative spatial map of change in HbT during forelimb activation.

### 2.4 Focal Cerebral Ischemia

To induce an occlusion in a distal pial branch of the MCA, while causing minimal damage to the surrounding parenchyma, we used a modified version of photothrombosis previously described by Watson et al.^19^ The 520-nm collimated laser diode was tuned to a postobjective power of 0.6 mW, which was shown to be sufficient to occlude the target vessel. The postobjective laser power was measured just below the focus before the start of each photothrombosis experiment. For illumination, the branch of the MCA supplying the forelimb region was identified using a response map obtained by intrinsic optical signal imaging during forelimb stimulation. Mice were head-fixed and positioned under the imaging system so that the illumination spot was on the target vessel. A translation stage was used for bulk positioning of the mouse and a kinematic steering mirror was used for fine changes to steer the laser beam to the target vessel. The vasculature was visualized under blue light with a multispectral camera. Since Rose Bengal is activated with green light, blue light was used to visualize the vasculature to prevent any unwanted photoactivation. Baseline CBF was obtained using LSCI for 10 min prior to the start of photothrombosis. Rose Bengal (100  μl, 15  mg/ml in saline) was injected retroorbitally while the mouse was anesthetized briefly with isoflurane. The active use of isoflurane, during induction and retroorbital injection, was limited to a minute. Isoflurane was immediately discontinued after injection and the mice were allowed to recover from anesthesia, as determined by a return to baseline blood flow in the real-time laser speckle contrast images as well as exhibiting natural behavior such as whisking, before performing photothrombosis.

One set of animals underwent traditional photothrombosis (nonoptimized protocol) where the target vessel was illuminated for 15 min, and the other set of animals underwent optimized photothrombosis (optimized protocol), where real-time laser speckle contrast feedback was used to guide the duration of illumination. In the optimized protocol, when the target vessel was occluded, as indicated by the branch disappearing during real-time LSCI, the 520-nm laser was decreased in power to half (0.3-mW postobjective) for 2 min and then turned off. If the branch reperfused, as indicated by a return in blood flow as seen in real-time LSCI, during the 1 h that the mice were monitored under LSCI, the 520-nm laser was turned on until the vessel was occluded again. Sufficient Rose Bengal was present in the blood stream over an hour after injection as shown in Fig. S1 in the Supplementary Material and can be in the blood stream for several hours after injection.^38^^,^^39^ Similar to other studies, we found it necessary to target and occlude collateral branches in addition to the main target vessel in order to stabilize the effects of the stroke, since collateral supply or even flow reversal can prevent a single vessel occlusion from having a significant reduction in blood flow in the target region.^26^^,^^40^ Collateral branches were determined on a case-by-case basis by observing flow redistribution in the MCA-supplied field using the real-time LSCI feedback.

To distinguish between a passive circulatory collapse or distal microembolism in the nearby capillary network closest to the illumination point and a direct capillary photothrombosis near the illumination area, we tested our protocol in an additional animal, to thrombose two separate pial veins with both the optimized and nonoptimized illumination strategies. Those targeted veins were of similar size to the previously targeted arterioles to allow comparison. When a cortical vein is thrombosed at a single focal point, we can still expect at least some degree of redistributed flow in the nearby upstream capillary bed close to the illumination area, which can be easily detected with optical coherence tomography (OCT) angiography. This way, we could avoid the effect of microemboli and a passive circulatory collapse, better revealing the directly photothrombosed zone.

### 2.5 Optical Coherence Tomography

OCT was used to obtain angiograms of the vasculature at the site of illumination before and after photothrombosis to analyze the vascular damage. A spectral domain OCT system (1310-nm center wavelength, bandwidth 170 nm, Thorlabs) was used for obtaining angiograms as described previously.^41^ Briefly, OCT-angiograms were obtained using a frame-to-frame subtraction of the repeated complex OCT B-scan signal. Static tissue will show no (or very low) difference between the repeated B-scans and will appear as dark regions in the OCT angiogram. On the other hand, moving red blood cells within a vessel will cause OCT signal fluctuations resulting in a large intensity difference between the repeated B-scans and will show up as bright areas in the OCT angiogram. A $600\times 600\;{\mu m}^{2}$ region around the target vessel was imaged before stroke and 1 h after stroke. The region was scanned with 400×400  pixels to obtain a pixel size of $1.5\times 1.5\;{\mu m}^{2}$. Each OCT angiogram was repeated 20 times to obtain an average of 20 angiograms. The transverse and axial resolutions of the OCT system using a 10× objective (Mitutoyo) were 3.5  μm×3.5  μm×3.5  μm.

### 2.6 Histological Analysis

In order to confirm the formation of an infarct, we performed histological staining with triphenyl-tetrazolium chloride (TTC) on fresh tissue at 24 h after photothrombosis on two mice. TTC staining is used to differentiate between metabolically active and inactive tissue and is commonly used to identify infarcted tissue. The mice were deeply anesthetized with isoflurane, decapitated, and their brains were removed for TTC staining. The intact brain was incubated at 37°C for 30 min in 2% TTC in 1×PBS. Images were acquired immediately after staining.

To assess the damage done to tissue at the illumination site, we stained for necrotic and apoptotic cells using propidium iodide (PI). PI is a fluorescent marker that binds to DNA of a cell but is not membrane permeable. This allows us to differentiate necrotic and apoptotic tissue from healthy cells. Mice were injected with 300  μl of 1  mg/ml of PI diluted in 300  μl of saline intraperitoneally 24 h after photothrombosis. We performed *in vivo* two photon imaging of PI 3 h after injection at the illumination site and the stroke core. The mice were perfused 4 h after PI injection with heparinized PBS followed by a 10% w/v gelatin solution in PBS with 30  mg/ml of FITC-albumin in order to visualize the vasculature during *ex vivo* imaging. The brains were extracted and transferred to 4% PFA for 6 h followed by 1×PBS for 3 days. The brains were then index matched with increasing concentration of fructose and sliced at the location of the target vessel for occlusion. The brains were then mounted in 100% fructose and imaged with a two-photon microscope to assess depth of tissue damage.

### 2.7 Behavioral Testing

The cylinder test was used in three mice to assess behavioral deficit in forelimb use over the course of 4 weeks following stroke.^42^^,^^43^ Baseline testing was obtained the week before stroke induction to assess basal preference in forepaw use. Following photothrombotic stroke mice were tested at 4 h, 1 day, 3 days, 1 week, 2 weeks, 3 weeks, and 4 weeks. Each testing session involved placing a mouse in a clear glass cylinder and videotaping its natural behavior from below for 15 min. Forelimb use was assessed by counting the number of times the mouse used each forelimb to make first contact with the cylinder wall during rears. Asymmetry in forelimb use after stroke was quantified as a percent change from baseline use of the contralateral forelimb. Change from baseline was used to compensate for the fact that some mice have a preference for one paw over the other even before a stroke.

### 2.8 Data Analysis

Flow dynamics were analyzed offline through spatial speckle contrast by applying the commonly used model of blood flow index.^44^ The speckle contrast was calculated from raw images captured by the camera using the following equation:

$$K=\;\frac{\sigma_{s}}{I},$$

where the spatial speckle contrast (K) is defined as the ratio of the spatial standard deviation ($\sigma_{s}$) and the mean intensity I of the pixels in a 7×7  bin. To provide a qualitative measure of blood flow dynamics, the blood flow index was calculated using^35^

$$\mathrm{BFI}=\;\frac{1}{K^{2}}.$$

OCT angiograms were analyzed for capillary density differences between the optimized and nonoptimized photothrombosis protocols. A custom MATLAB code was used to segment the vasculature and obtain the capillary density of a 150-μm-thick section of the $600\times 600\;{\mu m}^{2}$ region of interest. Capillary segmentation was performed below the large surface vessels. The mean and standard deviation of capillary density was compared before and after stroke for the two groups. In addition to mean capillary density, the capillary density as a function of radial distance from the illumination site was also calculated. Results are expressed as mean and standard deviations. Statistical analyses were made using an ANOVA with *post hoc* comparisons using t-tests. For capillary densities, a two-sample t-test was performed to compare densities before and after stroke for each protocols. Additionally, the two-sample t-test was used to compare the capillary densities at different radial distances between the optimized and nonoptimized protocols. For behavior analysis with the cylinder test, multiple pairwise comparisons were made with the pair-sample t-test at each time point with respect to baseline. A p-value of <0.05 was accepted as statistically significant. Individual p-values have been listed in Table S1 in the Supplementary Material.

## 3 Results

### 3.1 6-μm Spot is Superior to a 30-μm Spot in Preventing Capillary Damage as shown by Monte Carlo Simulations

The photothrombosis laser was incorporated into an existing LSCI system via epi-illumination to be able to simultaneously image changes to blood flow during vessel occlusion. To target a single branch of the MCA without damaging the surrounding parenchyma, we designed a laser beam path that resulted in the smallest spot size, given the imaging system’s parameters. Filling the back aperture of a 0.1-NA objective resulted in a spot size with a lateral resolution of 6  μm in diameter at the focus and a Rayleigh range of 60.8  μm. Zemax was used to design the required scan lens in order to fill the back pupil of the 2× objective with a 200-mm tube lens. The required beam profile was achieved with a combination of three planoconvex lenses that make up the scan lens. The Zemax model of the photothrombosis arm is shown in Fig. 1(b). The true beam size was validated and confirmed using a knife-edge beam profiler.

**Fig. 2:**
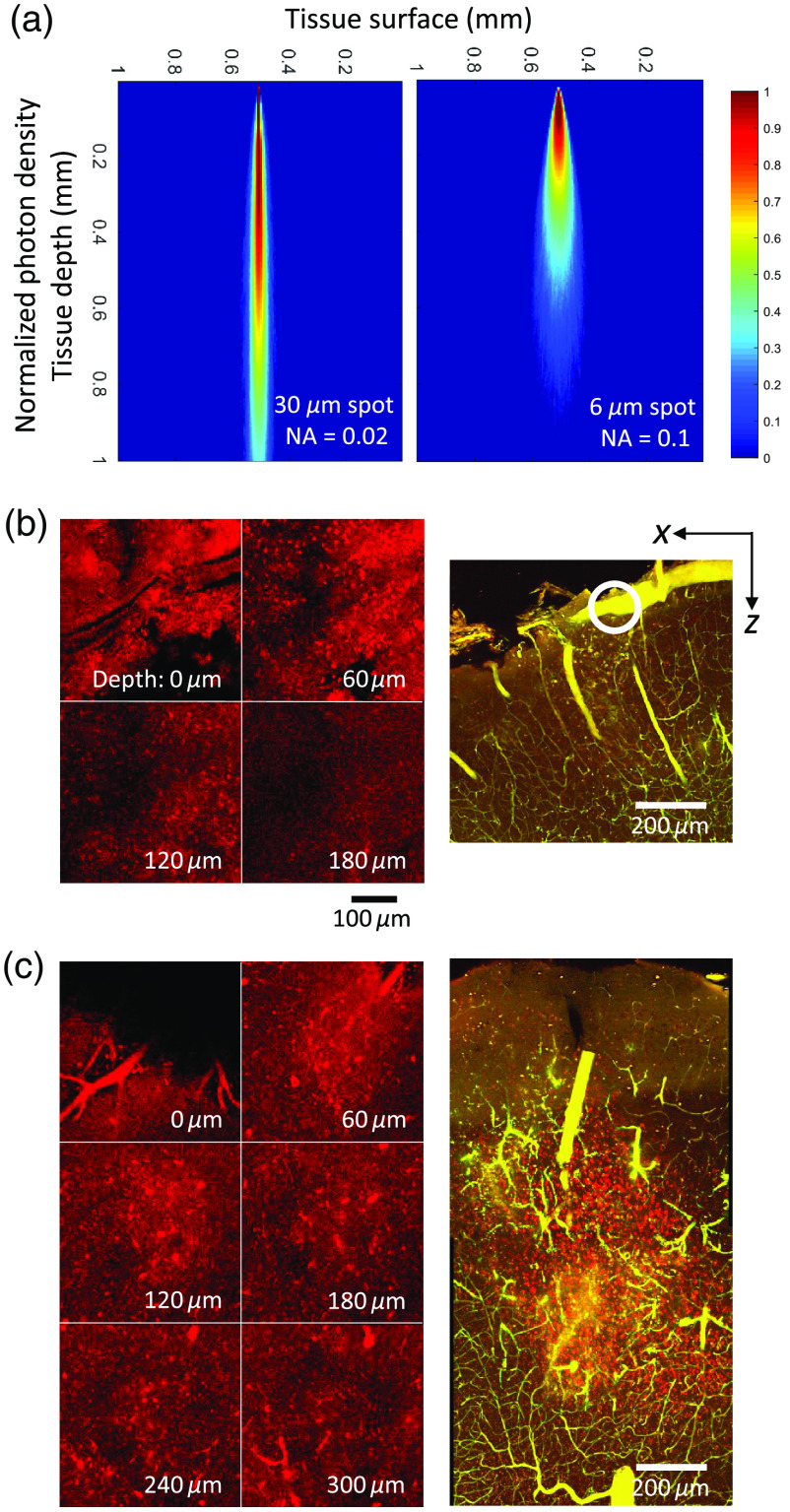
(a) Monte Carlo simulation of photon density for (left) a 30-μm spot with numerical aperture of 0.02 and (right) a 6-μm spot with numerical aperture of 0.1. (b) PI staining *in vivo* (left) and *ex vivo* (right) at the site of illumination (circled in white). (c) PI staining *in vivo* (left) and *ex vivo* (right) at the stroke core.

Photon migration within the tissue for the 6-μm spot was modeled using a Monte-Carlo simulation and compared against a simulation of a 30-μm spot size [Fig. 2(a)], since a number of previous studies have used a 30-μm spot to perform targeted photothrombosis. A 30-μm spot will have a very small NA of 0.02 and a Rayleigh range of 1.4 mm as shown in Fig. 2(a), resulting in less divergence of the beam. Thus power density falls off slowly for the 30-μm spot size, which will result in damage of not only the target vessel but also all the vessels within the parenchyma down to 1 mm. However, a 6-μm illumination spot has an NA of 0.1 and the beam diverges more quickly such that any parenchymal photothrombotic damage will be limited to ∼150  μm below the target vessel as the power density beyond that depth drops below the threshold for activating Rose Bengal.

Tissue damage in depth due to illumination with the 6-μm spot was validated *in vivo* and *ex vivo* using PI staining of damaged cells. Figure 2(b) shows damage at the target vessel location where the left panel indicates PI staining *in vivo* at 60-μm depths apart starting from the surface and the right panel shows damage *ex vivo*. PI positive cells are no longer visible at 180  μm below the surface, which was confirmed *ex vivo* where the damage was also limited to 150 to 200  μm below the surface. This was in contrast to damage observed at the stroke core [Fig. 2(c)], which was over 500  μm away from the illumination site and any damage was due to occlusion itself and not the illumination. PI positive cells are present even at 300  μm below the surface *in vivo* as shown in the left panel of Fig. 2(c) and the full extent of the stroke core is shown *ex vivo* in the right panel of Fig. 2(c) where it extends deep into cortex.

### 3.2 Optimized and Nonoptimized Methods Produce an Ischemic Stroke

**Fig. 3:**
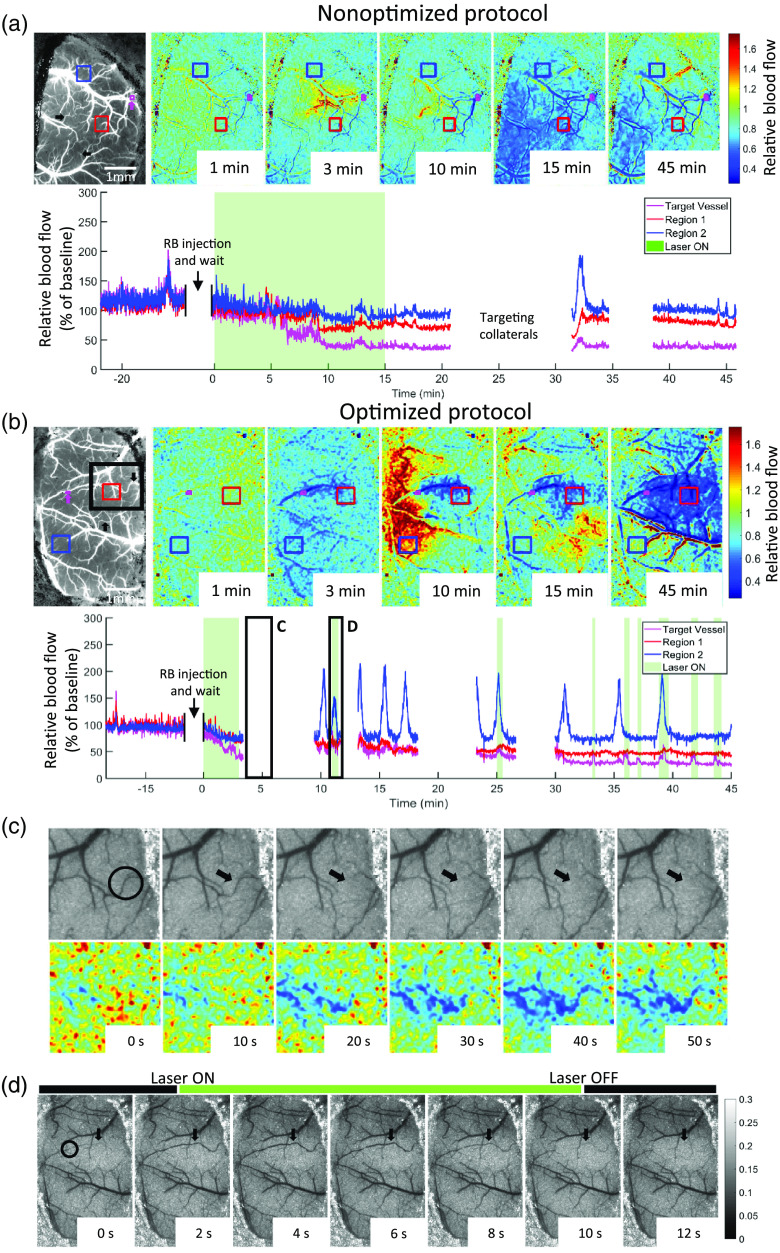
Relative CBF maps during photothrombosis. (a) Relative CBF during nonoptimized photothrombosis and (b) during optimized photothrombosis. Top panel shows spatial blood flow dynamics and bottom panel shows temporal dynamics for the regions specified in the top panel. Leftmost image in the top panel of (a) and (b) shows a reference indicating the target vessel in magenta arrows and collateral branches targeted for occlusion in black arrows. Both nonoptimized and optimized protocols show similar blood flow dynamics during occlusion. Green shaded region on the bottom panel corresponds to photothrombosis laser ON periods. (c) Representative images showing collateral occlusion (circled in black). Top panel shows laser speckle contrast images as visualized in real time. Bottom panel shows relative blood flow changes associated with the occlusion. The region shown is represented as the black square in the reference image of the top panel of (b) and the time course chosen for images in (c) is shown as the first black square indicated as “C” at the bottom panel of (b). (d) Representative images showing criteria for laser ON/OFF periods in the optimized protocol. The time course chosen for images is shown as the second black square indicated as “D” in the bottom panel of (b). Laser is turned on when the target vessel recanalizes, indicated with a drop in intensity in real-time laser speckle contrast, and turned off when the vessel is occluded again, indicated with an increase in signal intensity of the target vessel. (e) Video 1 showing real-time laser speckle contrast as seen during photothrombosis (left panel) and the associated blood flow changes (right panel). Since the mouse is being moved during collateral branch illumination, those segments have been removed for ease of visualization of the spatial dynamics observed during photothrombosis (Video 1, 13.6 MB, MP4 [URL: https://doi.org/10.1117/1.NPh.7.1.015005.1]).

Typically, previous studies have used a fixed duration of 10 to 15 min of laser illumination to occlude the target vessel.^20^^,^^39^^,^^45^ Following a similar protocol, in one group of animals, we illuminated our target vessel for 15 min with a laser power of 0.6 mW. We call this nonoptimized protocol. Occlusion was confirmed by measuring a greater than or equal to 80% drop in the qualitative blood flow index with LSCI and also a >50% decrease in the region supplied by the target vessel by monitoring real-time spatial LSCI feedback. For the optimized group, we used real-time LSCI feedback to guide the duration of illumination. Both the nonoptimized and optimized groups showed an 80% decrease from baseline CBF in the target vessel as seen in the time course of the relative blood flow changes in the bottom panel of Figs. 3(a) and 3(b). Additionally, the regions supplied by the target vessel showed similar patterns of change in CBF from baseline in both groups [Figs. 3(a) and 3(b)]. The stroke core, which is the area supplied by the target vessel and indicated by region 1, sees a 60% drop in blood flow in both groups. Region 2 is shown as a representative region selected away from the tissue supplied by the target vessel.

CBF analysis is not provided for the periods when the collaterals were targeted, as indicated by breaks in the CBF time course plots since we needed to reposition the mouse so that the desired vessel was directly under the illumination spot. The collaterals targeted are shown with black arrows in the leftmost reference images in Figs. 3(a) and 3(b). We found it necessary to target two collaterals on average to stabilize the effects of the stroke. An example collateral target is shown in Fig. 3(c) with the black circle. The top panel shows real-time laser speckle contrast images and the bottom panel shows the associated change in blood flow compared to baseline. We followed the same parameters of the optimized protocol to target the collaterals, where the illumination was turned on until the vessel was occluded following which the laser was turned off and the mouse repositioned to the original location. If the collateral branches recanalized, the mouse was moved again in order to occlude them. The time course of collateral occlusion is represented by breaks in the CBF time course in Figs. 3(a) and 3(b).

The optimized protocol used real-time laser speckle feedback to guide the duration of illumination of the target vessel. The green shaded region of the time course in Fig. 3 is when the 520-nm laser was on. After the initial occlusion period, the laser was turned on again when the target vessel recanalized, as shown by representative images in Fig. 3(d). A return of blood flow, indicated by a drop in laser speckle contrast signal intensity, was used as the criteria to turn the laser on, and a subsequent occlusion, indicated by an increase in signal intensity, was used to turn the laser off. This criteria for a return of blood flow and the subsequent occlusion are qualitative measures that are easily visualized in real-time laser speckle contrast images as the entire branch recanalized or disappeared, respectively. A video showing real-time laser speckle contrast and the associated CBF changes is included to better visualize the effects of photothrombosis (see Video 1).

### 3.3 Optimized Method is Superior to the Nonoptimized Method in Preventing Capillary Thrombosis

**Fig. 4:**
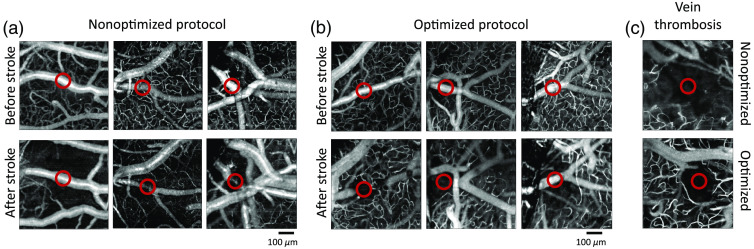
(a) OCT angiograms of flowing vessel before stroke (top panel) and 1-h after nonoptimized stroke (bottom panel). (b) OCT angiograms of flowing vessel before stroke (top panel) and 1-h after optimized stroke (bottom panel). (c) Vein thrombosis after nonoptimized stroke (top panel) and after optimized stroke (bottom panel). The target vessel is shown by the red circle.

The effect of photothrombosis on blood flow in the surrounding capillaries was assessed *in vivo* using OCT angiograms. The difference between image intensities of two repeated B-scans permits the detection of flowing capillaries. Figure 4 shows the average OCT angiograms of both the nonoptimized and optimized photothrombosis methods for three mice in each group. In Fig. 4(a), we show that after nonoptimized photothrombosis there is a significant decline in capillary density immediately surrounding the targeted pial artery compared to before stroke. The left panel in Fig. 4(a) also shows that 1 h after stroke, even though the surrounding parenchymal capillaries remained occluded, the target branch has recanalized. This indicates that the traditional method of illuminating the target vessel for a fixed longer duration is not necessarily sufficient to occlude the vessel for even 1-h poststroke, but has produced a persistent cessation of blood flow in the immediate vicinity. These capillaries have been photothrombosed because of the prolonged laser exposure in the nonoptimized method, and while the pial artery has recanalized in this case, the capillaries have not because of their smaller diameter stabilizing the thrombi in the capillaries.

Figure 4(b) shows the averaged OCT angiograms for the optimized protocol and we see that just the illuminated target vessel is occluded and all the surrounding capillaries are intact. Both the optimized and nonoptimized protocols show spontaneous recanalization in the target vessel after photothrombosis in some cases. Any drop in surrounding vessel density in the optimized protocol is due to block of capillaries downstream of the target vessel. This can be shown by thrombosing a vein as veins do not have any downstream capillaries and hence the capillary network should remain intact unless directly illuminated by the laser. Figure 4(c) shows a vein photothrombosis with the optimized and nonoptimized protocol. We see that with the optimized protocol only the target vessel is thrombosed and the capillary network within the immediate vicinity is intact, whereas in the nonoptimized protocol, the capillary network around the target vessel is also thrombosed. The optimized laser illumination ensures that the laser exposure to the capillaries remains below the threshold for photothrombosis to occur.

**Fig. 5:**
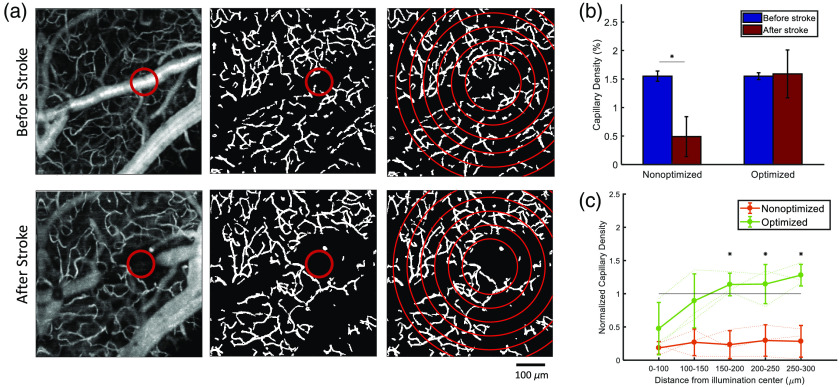
(a) Example schematic of vascular segmentation in the optimized protocol. Left column shows an example angiogram with occlusion site within the red circle. Middle column shows capillaries segmented beneath the larger pial vessels. Right column shows 50  μm concentric rings used to calculate capillary densities as a function of distance from the occlusion site. (b) Average vessel density for nonoptimized and optimized methods before and 1-h after stroke. The nonoptimized protocol shows a significant decrease in capillary density after stroke compared to before stroke (*p<0.05). (c) Capillary density as a function of distance from the occlusion site normalized to the baseline capillary density. Capillary density of the nonoptimized method was significantly less than that of the optimized method at distances >150  μm from the occlusion site (*p<0.05).

Quantitative analysis of vessel density also shows a decrease in capillary density for the nonoptimized photothrombosis, as seen in Figs. 5(b) and 5(c). Figure 5(a) shows an example OCT angiogram before and after stroke (left column), with the red circle indicating the site of laser illumination. The middle panel of Fig. 5(a) shows the maximum intensity projection of the segmented vasculature of the same example mouse for a 150-μm-thick section of capillaries. In order to look at the capillary density, as a function of distance from the site of occlusion we constructed 50  μm rings starting at 100  μm from illumination center and out to 300  μm. Figure 5(b) shows the capillary density for the full imaged region before and after stroke averaged across three mice for each group. The nonoptimized protocol shows a significant decrease in capillary density after stroke compared to before stroke (p<0.05), whereas the optimized protocol does not show a significant decrease in capillary density. When looking at the capillary density as a function of distance from illumination, we see that the capillary density of optimized protocol returned to baseline levels at 150 to 200  μm from the center as shown in Figure 5(c). However, the capillary density of the nonoptimized protocol remained at <50% of baseline vessel density out to 300  μm. Capillary density of the nonoptimized method was significantly less than that of the optimized method at >150  μm from the occlusion site (p<0.05).

### 3.4 Formation of a Stable Infarct was Confirmed Using Blood Flow Changes, Histological Validation, and Behavioral Analysis

**Fig. 6:**
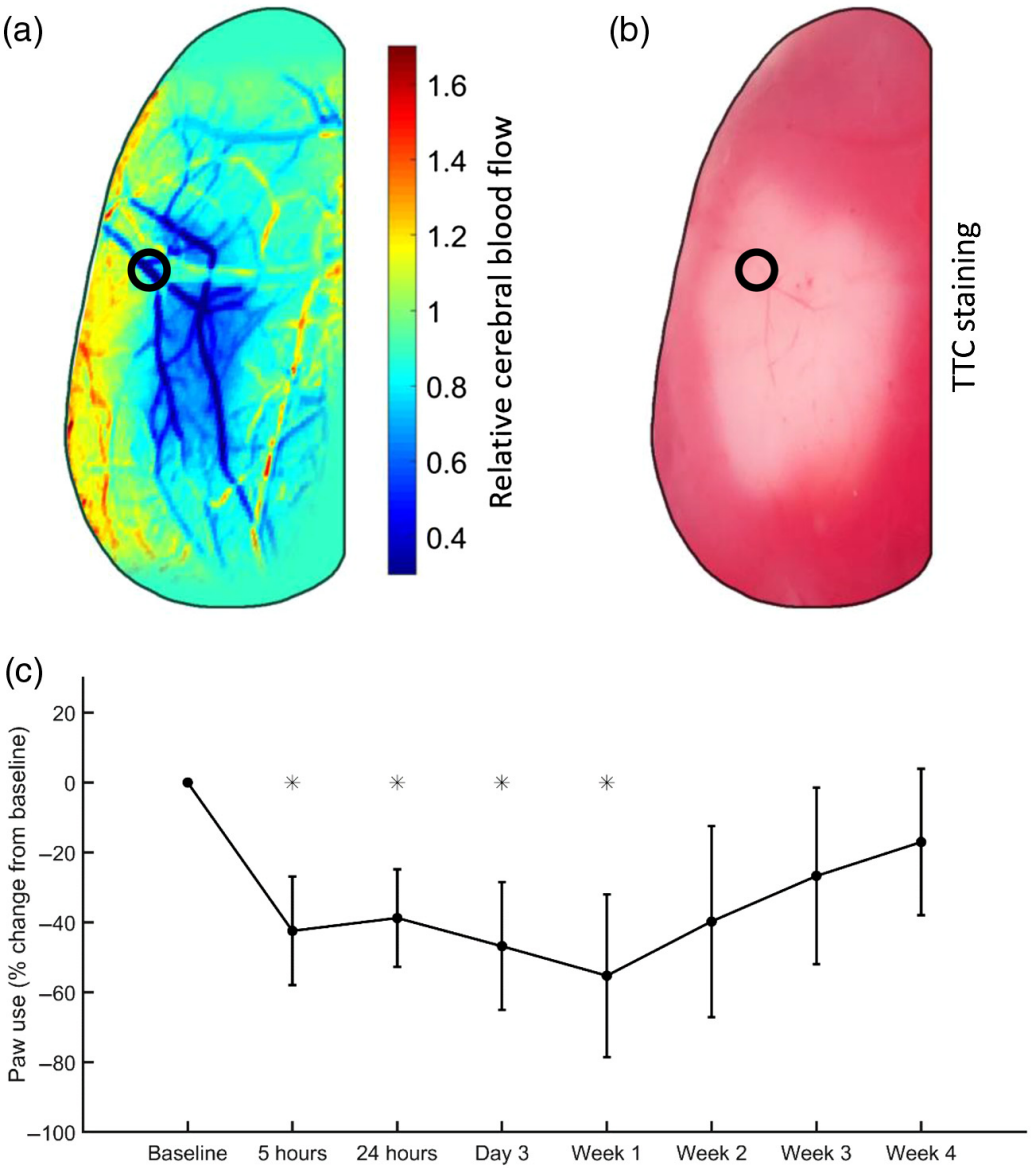
(a) Relative CBF at 1 h after photothrombosis. The target vessel is indicated with the black circle. (b) TTC stain at 24 h after photothrombosis. The infarct site coincides with the area of reduced CBF (<60% of baseline) shown in (a). (c) Percent change in paw use of the impaired forelimb (contralateral to stroke) over the course of 4 weeks. Mice showed a significant decrease in the use of the impaired forelimb specifically till 1 week following photothrombosis. *p<0.05.

In this study, we are capable of simultaneously monitoring changes to blood flow during stroke induction. This allows us to visualize and confirm in real time the occlusion of the target vessel as well as a drop in blood flow to the area supplied by the vessel. Figure 6(a) shows a representative image of relative CBF 1 h after photothrombosis. We observed a greater than 80% drop in blood flow within the target vessel and greater than 50% decrease in blood flow in the region supplied by the target vessel. TTC staining of viable tissue was used to confirm infarct formation at 24 h after photothrombosis in a subset of animals. Figure 6(b) shows infarcted tissue corresponding to the region of low blood flow in Fig. 6(a). The target vessel is marked with black circles. In addition to decreased blood flow and histological damage, we monitored change in contralateral forelimb use with the cylinder test at various time points following photothrombosis. We observed a decrease in use of the forelimb contralateral to the ischemic hemisphere (impaired forelimb) when compared to baseline use as shown in Fig. 6(c). There was a significant decrease (p<0.05) in the use of the impaired forelimb till one week after photothrombosis, following which behavior trended toward recovery to baseline. With these metrics, we can confirm that our optimized photothrombotic stroke model can produce a reliable infarct with associated functional deficits.

### 3.5 Chronic Use of Our Model Was Validated Using Longitudinal Imaging of Vasculature

**Fig. 7:**
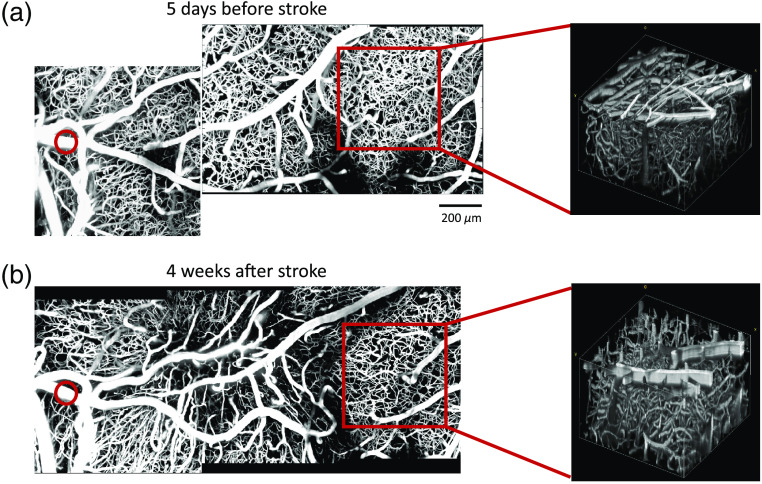
Two-photon maximum intensity projections (left) and volumes (right) of 400  μm stack (a) 5 days before photothrombosis and (b) 4 weeks after photothrombosis. Red circle indicates vessel targeted for photothrombosis. Red square indicates regions chosen for volume projections.

Our cranial window preparations allow longitudinal monitoring in awake mice, which can be used to study stroke recovery mechanisms or the long-term effects of therapies. Here we show that we are able to follow vascular structure using two-photon microscopy for one month following photothrombosis (Fig. 7). Although we followed the vasculature for one month, in which point the mice were sacrificed for *ex vivo* analyses, the mice can be monitored for even longer time points if desired since one-month poststroke showed minimal deterioration to the window quality. An inflammatory response that results due to the lesion could cause cellular build up under the window which could deteriorate the quality of the window. However, that deterioration is minimal and we are able to penetrate to 400  μm below the surface with sufficient signal-to-background ratio. In order to test the longevity of the cranial windows, we have followed control mice for 6 months and expect that the stroked mice can also be followed for that long. Thus our model is ideal for longitudinal stroke studies and can be used to study various physiological parameters across a number of imaging modalities.

## 4 Discussion

We have developed a chronic animal model that can be used to induce ischemic stroke in awake mice while monitoring changes to CBF. We also show that the limited laser spot size and real-time CBF permits us to occlude a target pial artery without occluding the surrounding and underlying capillaries. Although a number of previous models have performed single-vessel occlusion and produced the desired ischemic core and peri-infarct zone, they were performed on anesthetized mice.^25^^,^^26^^,^^28^^,^^46^ Anesthetics are known to modulate blood flow, which could impact the mechanisms leading to parenchymal failure after a stroke.^12^^–^^14^^,^^47^ Additionally, it has been shown that anesthetics can mask the benefits of a neuroprotective therapy.^20^ A study has shown the ability to use an MCA occlusion model without the effects of anesthesia, but the animal preparation is acute and does not allow the animals to recover from surgical procedures, which could be a confounding factor.^15^ Our model uses a cranial window preparation that is performed three weeks prior to stroke induction, allowing the mouse time to recover from the surgery. Additionally, our cranial windows allow longitudinal monitoring of stroke recovery mechanisms, which can be used to study long-term stroke progression or the long-term effects of therapies.

The photothrombotic stroke model is advantageous for studying the mechanisms of the functional, structural, and behavioral responses involved in stroke recovery due to its ability to precisely target a desired region.^21^^–^^24^^,^^48^ However, a drawback of directly targeting an anatomically distinct area, which includes larger vessels and parenchyma, using photothrombosis, is the relatively small ischemic penumbra that results due to oxidative damage primarily within the ischemic core, rather than gradually distributed in the peri-infarct zone.^5^^,^^6^^,^^25^ This hinders the understanding of how mechanisms within the peri-infarct influence functional recovery. In this paper, we have shown that targeting an upstream branch of the MCA supplying a functionally distinct area is capable of creating an infarct core in the area it supplies as seen by >60% drop in blood flow, as well as a penumbra as seen by a 40% to 60% drop in blood flow in the area surrounding the ischemic core. This method of artery targeted photothrombosis that leads to a widened vascular penumbra suitable to study recovery mechanisms within the peri-infarct zone has also been shown previously by Clark et al.^25^ We have also shown in our present study that our model is capable of targeting a distinct functional area of the forelimb representation that produces longitudinally quantifiable deficits in forelimb use.

Additionally, through Monte Carlo simulations, we have shown that the size of the beam used to occlude the target vessel must be considered in order to minimize unwanted photodamage to the surrounding parenchyma. We have shown that it is not sufficient to decrease the lateral resolution of the illumination spot to the size of the branch being targeted. The size of a distal MCA is ∼30 to 50  μm, and a spot size with a lateral resolution of 30  μm will result in vascular damage of over 1 mm beneath the target vessel. Decreasing the spot size to a 6-μm lateral resolution resulted in significantly less unwanted vascular damage and was limited primarily to the target surface vessel. Histological evidence validated that the parenchymal damage was limited to the surface at the site of illumination, whereas the core showed damaged parenchyma deep in cortex.

Other groups have shown the ability to use photothrombosis to induce the desired core and peri-infarct zones using variations of the vessel occlusion model. A number of single-vessel occlusion models have been proposed that utilize an objective with higher magnification and larger numerical aperture to target a region smaller than the vessel itself to minimize photodamage.^26^^,^^46^ It typically involves starting laser illumination on one side of the vessel and traversing the laser beam laterally to facilitate thrombus formation along the vessel. Although this leads to negligible unwanted photodamage, it does not allow simultaneous macroscopic imaging of CBF. These models also result in relatively small ischemic regions that are useful for studying the effects of small strokes but are not large enough to follow functional recovery mechanisms. Another model is artery-targeted photothrombosis using a digital micromirror device to provide patterned illumination along the length of the artery with minimal exposure to the surrounding parenchyma.^21^^,^^25^^,^^49^^,^^50^ This method also allows targeting multiple arteries simultaneously, permitting illumination of collaterals together with the main branch and thus controlling perfusion within the area. Some drawbacks of these methods are that photothrombosis is still performed under anesthesia and the instrumentation is relatively more expensive and difficult to implement.

In addition to spatially adjusting the laser illumination, we have also shown the advantages of temporally controlling the duration of illumination. A previous study has mentioned the use of real-time blood flow feedback to guide photothrombosis,^27^ and in this study we show that the typically used fixed duration illumination protocol results in unwanted vascular damage and optimizing that protocol to guided illumination using real-time blood flow feedback further minimizes damage to the surrounding parenchyma. The duration of illumination is limited to when the target vessel is flowing as observed using LSCI. Capillary damage was analyzed by looking at the density of flowing capillaries obtained from OCT, where we show an intact flowing capillary network in the immediate vicinity of the occluded vessel in the optimized protocol as opposed to the nonoptimized protocol. To rule out the possibility of downstream capillary thrombosis due to laser illumination, we performed optimized thrombosis on a vein, which did not show any drop in capillary density apart from the target branch. However, vein photothrombosis using the nonoptimized protocol showed a similar drop in capillary density compared to artery illumination with the nonoptimized protocol.

This photothrombosis stroke model also has a few limitations. One limitation is the brief use of isoflurane during intravenous Rose Bengal injection. Although the use of isoflurane is limited to a minute, it must be noted that there may be remnants of its effects even after the discontinuation of anesthesia. As we are primarily concerned about the effects of isoflurane on blood flow during photothrombosis, our criteria of recovery is the return of blood flow to baseline levels as seen with real-time LSCI before the start of photothrombosis. A second limitation is that the laser needs to be manually turned on and off to optimize the illumination duration. This can be automated in the future by linking the real-time CBF from LSCI to the laser for photothrombosis. Additionally, the mouse needs to be laterally translated when targeting the collateral branches. This can also be improved upon by allowing a larger range of lateral control of the laser beam.

In brief, this model is fairly easy and relatively inexpensive to implement and can be used for experiments that more accurately mimic the mechanisms and recovery processes of ischemic stroke and allows longitudinal monitoring of recovery mechanisms.
