# Supplementary material for: Awake chronic mouse model of targeted pial vessel occlusion via photothrombosis
Source: Neurophotonics. 2020 Jan 30;7(1):015005. doi: 10.1117/1.NPh.7.1.015005 (PMC6992450; doi:10.1117/1.NPh.7.1.015005)
Supplement: Supplementary file 1 [file NPh_007_015005_SD001.docx]

**Supplementary Material**

1. **Supplementary Figures**


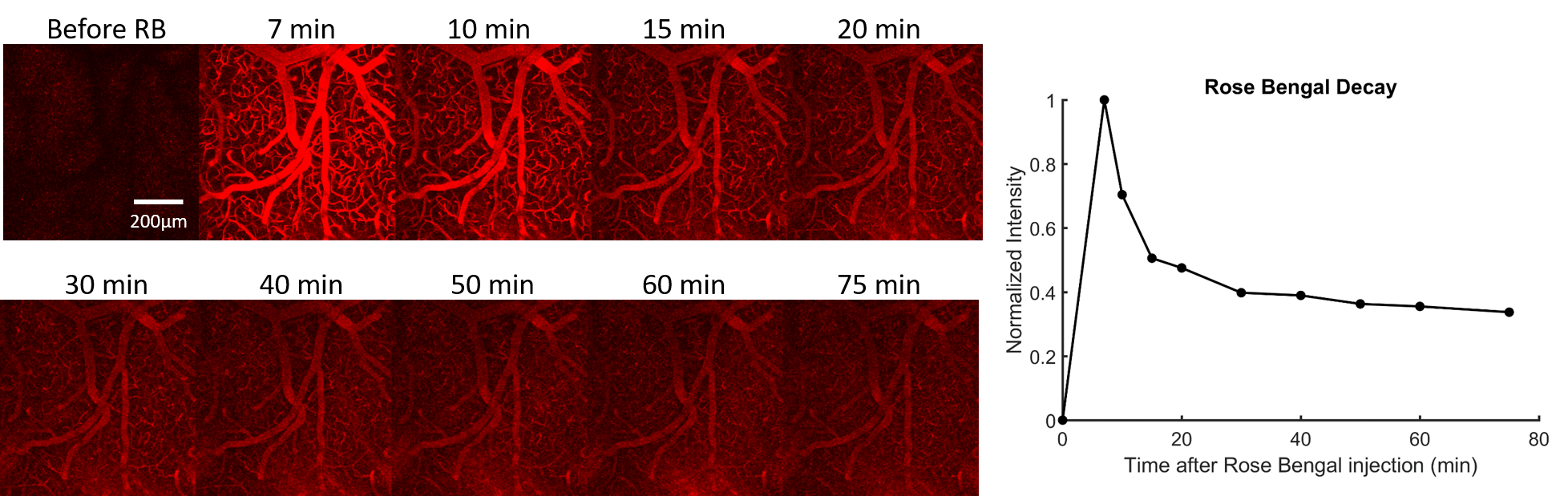


**Supplementary Figure 1:** Rose Bengal decay imaged using two-photon microscopy. The left panel shows 150 µm maximum intensity projections of the vasculature imaged before and at various time points after Rose Bengal injection. The laser and signal amplification parameters were kept constant throughout the session. The right panel shows normalized signal intensity over time. Rose Bengal decays exponentially as expected. We observe that ~40% of the initial Rose Bengal is still present in circulation at 1 hour after injection.

1. **Supplementary Tables**

**Supplementary Table 1**

Table of p-values from results of statistical tests. Significance is indicated with an asterisk.

**Supplementary Table 2**

Parts list for the combined photothrombosis, laser speckle contrast imaging, and intrinsic optical signal imaging system.

| **Combined multispectral and laser speckle imaging system** | | | | |
| --- | --- | --- | --- | --- |
| **Item** | | **Vendor** | **Part** | **Quantity** |
| **Illumination (IOSI)** | LED | Thorlabs | M470L3 | 1 |
|  |  | Thorlabs | M530L3 | 1 |
|  |  | Thorlabs | M625L3 | 1 |
|  | Filters | Thorlabs | FB470-10 | 1 |
|  |  | Thorlabs | FB530-10 | 1 |
|  |  | Thorlabs | FB620-10 | 1 |
|  | LED power | Thorlabs | LEDD1B | 3 |
|  |  | Thorlabs | KPS101 | 3 |
|  | Collimation | Thorlabs | ACL2520U-DG6-A | 3 |
|  |  | Thorlabs | SM1V05 | 3 |
|  |  | Thorlabs | SM1L03 | 3 |
|  | Dichroics | Thorlabs | MD568 | 1 |
|  |  | Thorlabs | DMLP490R | 1 |
|  |  | Thorlabs | DFM1B | 2 |
| **Illumination (LSCI)** | Laser diode | Thorlabs | LP785-SAV50 | 1 |
|  | Current controller | Thorlabs | LDC205C | 1 |
|  | Temperature controller | Thorlabs | TED200C | 1 |
|  | Laser diode mount | Thorlabs | LDM9LP | 1 |
|  | Collimation and expansion | Thorlabs | F280FC-780 | 1 |
|  |  | Thorlabs | AD1109F | 1 |
|  |  | Thorlabs | GBE05-B | 1 |
| **Objective** | 2X, 0.1NA, 56mm WD | Thorlabs | TL2X-SAP | 1 |
| **Image splitting** | Beamsplitter | Semrock | FF640-FDi02-t3 | 1 |
| **Filters** | IOSI | Thorlabs | FESH0650 | 1 |
|  | LSCI | Thorlabs | FB780-10 | 1 |
| **Camera lenses** | IOSI | Thorlabs | TTL200-A | 1 |
|  | LSCI | Thorlabs | TTL200-B | 1 |
| **Camera** | IOSI | Hamamatsu | ORCA Flash 4.0 V3 | 1 |
|  | LSCI | Basler | acA2040-90um NIR | 1 |
| **Translation stages** | X,Y translation | Thorlabs | LTS150 | 2 |
|  | Z translation | Thorlabs | MLJ150 | 1 |
| **Optomechanics** | Base breadboard | Thorlabs | MB30 | 1 |
|  | Microscope body | Thorlabs | CEA1400 | 1 |
|  | Objective holder | Thorlabs | ZFM1020 | 1 |
|  | Breadboad top | Thorlabs | CSA3010 | 1 |
|  | National instruments DAQ | NI | PCIe-6321 X Series DAQ | 1 |
| **Photothrombosis** | | | | |
| **Item** | | **Vendor** | **Part** | **Quantity** |
| Illumination | Laser diode | Thorlabs | L520P50 | 1 |
|  | Collimation | Thorlabs | C110TMD-A | 1 |
|  |  | Thorlabs | LDH56-P2 | 1 |
|  | Strain relief cable | Thorlabs | SR9HA-DB9 | 1 |
|  | Beam expander | Thorlabs | GBE05-A | 1 |
|  | Current controller | Thorlabs | LDC205C | 1 |
| Optics | Kinematic mirror | Thorlabs | KCB2C | 1 |
|  |  | Thorlabs | BB2-E02 | 1 |
|  | Scan lenses | Edmund Optics | 32-861 | 1 |
|  |  | Edmund Optics | 32-865 | 2 |
|  | ND filter | Edmund Optics | 46-123 | 1 |
|  |  | Thorlabs | LC6W | 1 |
| Optomechanics | Breadboard | Thorlabs | MB1224 | 1 |
|  | Lens housing | Thorlabs | SM2NR1 | 1 |
